# The cultivation conditions affect the aggregation and functionality of β‐cell lines alone and in coculture with mesenchymal stromal/stem cells

**DOI:** 10.1002/elsc.202100168

**Published:** 2022-05-20

**Authors:** Florian Petry, Denise Salzig

**Affiliations:** ^1^ Institute of Bioprocess Engineering and Pharmaceutical Technology University of Applied Sciences Mittelhessen Giessen Germany

**Keywords:** 3D cell culture, β‐cells, coculture, mesenchymal stromal/stem cells, spheroids

## Abstract

The manufacturing of viable and functional β‐cell spheroids is required for diabetes cell therapy and drug testing. Mesenchymal stromal/stem cells (MSCs) are known to improve β‐cell viability and functionality. We therefore investigated the aggregation behavior of three different β‐cell lines (rat insulinoma‐1 cell line [INS‐1], mouse insulinoma‐6 cell line [MIN6], and a cell line formed by the electrofusion of primary human pancreatic islets and PANC‐1 cells [1.1B4]), two MSC types, and mixtures of β‐cells and MSCs under different conditions. We screened several static systems to produce uniform β‐cell and MSC spheroids, finding cell‐repellent plates the most suitable. The three different β‐cell lines differed in their aggregation behavior, spheroid size, and growth in the same static environment. We found no major differences in spheroid formation between primary MSCs and an immortalized MSC line, although both differed with regard to the aggregation behavior of the β‐cell lines. All spheroids showed a reduced viability due to mass transfer limitations under static conditions. We therefore investigated three dynamic systems (shaking multi‐well plates, spinner flasks, and shaking flasks). In shaking flasks, there were no β‐cell‐line‐dependent differences in aggregation behavior, resulting in uniform and highly viable spheroids. We found that the aggregation behavior of the β‐cell lines changed in a static coculture with MSCs. The β‐cell/MSC coculture conditions must be refined to avoid a rapid segregation into distinct populations under dynamic conditions.

**Abbreviations**
ad‐MSC: adipose‐derived mesenchymal stromal/stem cell
1.1B4: a cell line formed by the electrofusion of primary human pancreatic islets and PANC‐1 cells
hMSC‐TERT: human mesenchymal stromal/stem cells immortalized with reverse transcriptase telomerase
INS‐1: rat insulinoma‐1 cell line
MIN6: mouse insulinoma‐6 cell line
MSC: mesenchymal stromal/stem cell

## 1 INTRODUCTION

The loss or dysfunction of pancreatic β‐cells is the main cause of diabetes, which has prompted research into the restoration of β‐cell mass in vivo. Optimal functionality (establishment of glucose homeostasis) is offered by the transfer of islets or primary β‐cells. Primary β‐cells fail to propagate in vitro and lose their functionality due to the harsh conditions during isolation and the lack of an authentic 3D environment [1, 2]. The reconstitution of a 3D environment that mimics in vivo conditions, such as the formation of spheroids, improves the viability and proliferation of β‐cells, stabilizes their developmental fate, and most importantly enhances the glucose‐dependent insulin secretion [3, 4, 5]. Human induced pluripotent stem cells (iPSCs) are proposed to overcome the limitations of a donor‐based diabetes therapy, as they can be expanded extensively, and can be transferred to patients without immunosuppression [6, 7]. However, the application of iPSCs requires complex and laborious differentiation protocols, and the resulting cells still lack in functionality [8]. To use isolated primary β‐cells and insulin‐producing iPSCs in a larger group of patients, scalable aggregation techniques are required to provide enough functional spheroids within a desired size range, and with a narrow size distribution. The mean diameter of native pancreatic islets is ∼150 μm [9], and it may therefore be beneficial to form spheroids of a similar size.

To gain more insight in the aggregation behavior of spheroids, we used three different β‐cell lines (human a cell line formed by the electrofusion of primary human pancreatic islets and PANC‐1 cells [1.1.B4], mouse insulinoma‐6 cell line [MIN6], and rat insulinoma‐1 cell line [INS‐1]) to investigate spheroid formation and growth, aiming at the production of homogenous, viable, and functional β‐cell spheroids. These cells can be used as models to understand the aggregation process, but we are aware that such cells will never be transferred to humans and are only suitable for diabetes research ex vivo. However, they are good models because the rodent β‐cell lines have a high expansion capacity, but maintain a moderate insulin secretion even as monolayers, whereas human β‐cell lines, such as 1.1B4, secrete 10–100 times less insulin than rodent cell lines [10, 11]. Using these three model β‐cell lines, we first tested standard static aggregation techniques, based on the aggregation of the cells in a cell‐repellent environment. As these systems are not scalable, we transferred the aggregation process to dynamic spinner and shaking flask systems, and compared the static and dynamic aggregation methods in terms of spheroid numbers, growth, viability, size, circularity, and functionality. Although the aggregation of these cell lines has been investigated before [3, 12, 13], we sought to compare them in a systematic manner, to identify both the cell‐dependent and technique‐dependent differences in their aggregation behavior.

**PRACTICAL APPLICATION**

Our systematic investigation of the static aggregation in different β‐cell lines and mesenchymal stromal/stem cells revealed differences in key properties, such as aggregation behavior, spheroid size, and connected mass transfer limitations, ultimately leading to a lower viability. In order to develop a scalable production process for defined insulin‐producing spheroids, we transferred the cells to three dynamic systems: shaking multi‐well plates, spinner flasks, and shaking flasks. This allowed us to overcome mass transfer limitations, but resulted in new challenges, such as heterogeneous power input and uncontrolled aggregation. Using baffled shaking flasks, we achieved a cell aggregation under isotropic fluid conditions. This enabled the production of defined aggregates that can be evaluated for a large‐scale manufacturing of insulin‐producing spheroids as a form of diabetes cell therapy, and for drug development and testing.

In addition to the provision of a 3D environment, the functionality, and viability of β‐cells can also be improved by a coculture with mesenchymal stromal/stem cells (MSCs) [14, 15, 16]. The coculture of MSCs with pancreatic β‐cells in vitro induced a prolonged, high‐level insulin secretion, and an improved survival of the pancreatic islets [14, 15, 16]. To our best knowledge, the coculture of different β‐cell lines and MSC types has not been compared under the same experimental conditions before. We investigated the influence of primary adipose‐derived mesenchymal stromal/stem cells (ad‐MSCs) and/or the immortalized cell line human mesenchymal stromal/stem cells immortalized with reverse transcriptase telomerase (hMSC‐TERT), which expresses telomerase reverse transcriptase [17], by coculturing them with the three β‐cell lines. As stated above for the monospheroids, we screened for suitable aggregation methods that generate stable and functional heterospheroids with different β‐cell/MSC ratios, with a focus on their viability and glucose‐dependent insulin secretion. Finally, we investigated spheroid formation in dynamic culture systems to define their aggregation behavior, viability, and functionality, compared to static aggregation methods.

## 2 MATERIALS AND METHODS

### 2.1 Cell cultures and culture media

Pre‐cultures of each cell type were tested for mycoplasma contamination (MynoxGold, Biochrom, W 10‐0200). The β‐cell lines INS‐1 (kindly provided by Sebastian Hauke, European Molecular Biology Laboratory) and 1.1B4 (MERCK, formerly Sigma–Aldrich, 10012801‐1VL) were cultivated in Roswell Park Memorial Institute (RPMI‐1640, Biochrom, FG 1385) medium supplemented with 10% fetal calf serum (FCS, Biochrom, S0615) and, for the INS‐1 cells, also 0.05 mM 2‐mercaptoethanol. MIN6 cells (Hölzel Diagnostika/Addexbio, C0018008) and hMSC‐TERTs (kindly provided by Prof. M. Kassem [17]) were cultivated in Dulbecco's modified Eagle's medium (DMEM, Biochrom, F 0415 and F 0425) supplemented with high glucose (HG, 4.5 g L^–1^) or low glucose (LG, 1 g L^–1^), respectively. The MIN6 culture medium (DMEM‐HG) was supplemented with 15% FCS, 2 mM glutamine (Bio&Sell, BS.K 0283) and 0.05 mM 2‐mercaptoethanol, whereas the hMSC‐TERT culture medium (DMEM‐LG) was supplemented with 10% FCS and 2 mM glutamine. The primary ad‐MSCs (PromoCell, C‐12977) were cultured in MSC Growth Medium 2 (MSC GM2, PromoCell, C‐28009B) and the recommended Supplement Mix (PromoCell, C‐39809).

The following seeding densities were used for each cell line/type: MIN6 = 8·10^4^ cells cm^2^, INS‐1 = 5 × 10^4^ cells cm^2^, and 1.1B4, hMSC‐TERT and ad‐MSCs = 8 × 10^3^ cells cm^2^. The cells were detached using trypsin (Biowest, L0940‐100) at 80% confluence, and were cultured at 37°C in an atmosphere of 5% (RPMI and MSC GM2) or 8% (DMEM LG/HG) CO_2_.

For the coculture of β‐cells with hMSC‐TERTs or ad‐MSCs, the β‐cell medium was used, and both MSC types were pre‐adapted by one passage in the β‐cell medium before coculturing.

### 2.2 Long‐term cell labeling

Before spheroid formation, β‐cells were stained with 7.5 μM 5‐(and 6)‐carboxyfluorescein diacetate, succinimidyl ester (CFSE) from the Cell Proliferation Kit I (PromoKine, PK‐CA707‐30050), according to the manufacturer's recommendations. The MSCs were stained with 10 μM Violet Proliferation Dye 450 (VPD450, BD Biosciences, 562158), according to the manufacturer's recommendations.

### 2.3 Static spheroid formation

We used 96‐well U‐bottom plates with cell‐repellent surfaces (Cellstar by Greiner Bio‐One, 650979) for static spheroid formation. The spheroids were prepared at eight different β‐cell/MSC ratios (0:1, 1:0, 1:1, 2:1, 4:1, 8:1, 12:1, and 16:1), each as 12 biological replicates. The cells were seeded, depending on the ratio, to achieve a total of 1000 cells per well and a culture medium working volume of 200 μL. After seeding, the cells were centrifuged at 300 g for 5 min, and incubated at 37°C in a 5% (INS‐1 and 1.1B4, RPMI‐1640) or 8% (MIN6, DMEM‐HG) CO_2_ atmosphere. We replaced 50% of the medium every other day.

### 2.4 Analytics of static spheroid formation

The spheroid samples were analyzed using a Cytation 3 cell‐imaging multi‐mode reader and Gen 5 v2.07.17 software (BioTek) to determine the spheroid count, size, and circularity. The cells were maintained at 37°C in the appropriate CO_2_ atmosphere (Subsection 2.3) during the analysis. The diameter (*Ø*) of spheroids in the static culture was measured every day for 7 days. In contrast to the standard procedure for the assessment of the growth rate [18] or the one‐dimensional representation of the diameter, the volume‐based growth rate *μ*_Vol_ of the spheroids provides the real 3D growth of the spheroids [19, 20]. This requires an increase in volume in the radial direction, due to the exponential growth of the cells in the outer layer of the spheroids. Furthermore, a constant volume of the cells is assumed over the course (Equation 1):

(1) $$\mu_{\mathrm{Vol}}=\frac{\ln\left(V_{\mathrm{Sph},2}\right)-\ln\left(V_{\mathrm{Sph},1}\right)}{t_{2}-t_{1}}\;$$

where *V*_Sph,1/2_ represents the spheroid volume at time point t_1/2_.

The resulting minimal time for doubling of the volume *t*_D,Vol_ was calculated as follows (Equation 2):

(2) $$t_{D,\mathrm{Vol}}=\frac{\ln\left(2\right)}{\mu_{\mathrm{Vol}}}\;$$

The spheroids were stained with 10 μM calcein‐AM and 10 μM ethidium homodimer‐1, incubated for 30 min at room temperature, and analyzed using the imaging fluorescence mode of the Cytation 3. As spheroids are 3D structures, we wanted to assess the “3D viability” instead of measuring a 2D area. Therefore, we calculated the volumes of the living zones and the whole spheroid (*V*_green_ and *V*_total_), and determined the viability of the spheroids using Equation (3):

(3) $$\mathrm{Viability}\;=\frac{V_{\mathrm{green}}}{V_{\mathrm{total}}}\;*100$$

### 2.5 Dynamic spheroid formation

Initial dynamic spheroid formation experiments were performed in 12‐well plates with suspension surfaces (Sarstedt, 83.3921.500). The working volume of the wells was 1 mL, and the seeding density was 0.5 × 10^6^ cells mL^–1^. After seeding, the plates were placed on a Celltron shaking platform (Infors) at 90 rpm within an incubator at 37°C in the appropriate CO_2_ atmosphere (Subsection 2.3) for 5 days.

We scaled up the dynamic spheroid formation in 250‐mL spinner flasks with magnetic pendulums (Integra Bioscience) and a working volume of 50 mL. The spinner flasks were seeded with 0.5 × 10^6^ cells mL^–1^, placed within an incubator at 37°C in the appropriate CO_2_ atmosphere (Subsection 2.3), and agitated at 35 rpm for 5 days.

The third dynamic culture system for spheroid formation comprised 100‐ml shaking flasks with an inner diameter of 0.064 m, four baffles, and a working volume of 20 mL. The flasks were placed on the Celltron shaking plate with an eccentricity of 2.5 cm within an incubator at 37°C in the appropriate CO_2_ atmosphere (Subsection 2.3). The flasks were seeded with 0.5 × 10^6^ cells mL^–1^ and agitated at 100 rpm for 5 days.

### 2.6 Analytics of dynamic spheroid formation

For the analysis of spheroids sampled from shaking flasks, 100‐μ aliquots were stained with 10 μM calcein‐AM and 10 μM ethidium homodimer‐1 in flat‐bottom 96‐well plates, incubated for 30 min at room temperature and analyzed using the Cytation 3. The number of spheroids we analyzed in the size range 20–300 μm depended on the cell type and aggregation behavior and was *n* > 400 for the INS‐1 cells, *n* > 1000 for the 1.1B4 cells, and *n* > 5000 for the hMSC‐TERTs. We set our threshold at 20 μm to discriminate between single cells and spheroids, meaning that all particles <20 μm were counted as single cells, and all particles ≥20 μm were counted as spheroids. The analysis of the dynamic spheroid formation in shaking flasks was based on three biological replicates (*n* = 3) with each sample measured as three technical replicates. Based on the green and red fluorescence, we measured the count, size, area, and circularity of the viable and the dead spheroids and single cells. We determined *μ*_Vol_ and *t*_D,Vol_, using Equations (1) and (2). We used the Sauter diameter *d*_32_ to describe the spheroid size distribution. The viability of the spheroids was calculated using Equation (3).

### 2.7 Glucose‐dependent insulin secretion assay

We measured 10 spheroids from the static cultures, or 1‐mL samples from each shaking flask with the corresponding number of spheroids. The samples were washed twice with phosphate‐buffered saline (PBS) to remove any residues of FCS, which might contain insulin. The spheroids were then incubated in DMEM (without FCS) containing 1.1 mM glucose for 40 min at 37°C, followed by DMEM (without FCS) containing 16.7 mM glucose under the same conditions. Following incubation, the supernatant was transferred to Eppendorf tubes with low‐protein‐binding surfaces, centrifuged (300 *g*, 5 min), and stored at −20°C. The insulin in the supernatants was measured in duplicates, using the corresponding enzyme‐linked immunosorbent assay (ELISA) kit: the human ultra‐sensitive insulin ELISA (EIA‐2337) for 1.1B4 cells, the rat insulin ELISA (EIA‐2049) for INS‐1 cells, and the mouse insulin ELISA (EIA‐3439) for MIN6 cells (all kits from DRG Instruments).

### 2.8 Statistical analysis

The results are presented as means ± standard deviations (STDV) of at least three independent experiments, if not stated otherwise. If relevant, statistical significance was determined, using the Student's *t*‐test. Statistically significant differences were indicated as follows: *p < 0.05, **p < 0.01, and ***p < 0.001.

## 3 RESULTS AND DISCUSSION

### 3.1 The characterization of spheroids from β‐cell lines

The three β‐cell lines grew moderately in vitro as monolayers while retaining a certain degree of functionality. Although monolayer data for these β‐cell lines have already been published (Supporting Information Table S1), we determined our own parameters as a benchmark to characterize the aggregation behavior of the cells in more detail (Supporting Information Table S1 and Figure S1).

#### 3.1.1 Static spheroid formation

**FIGURE 1:**
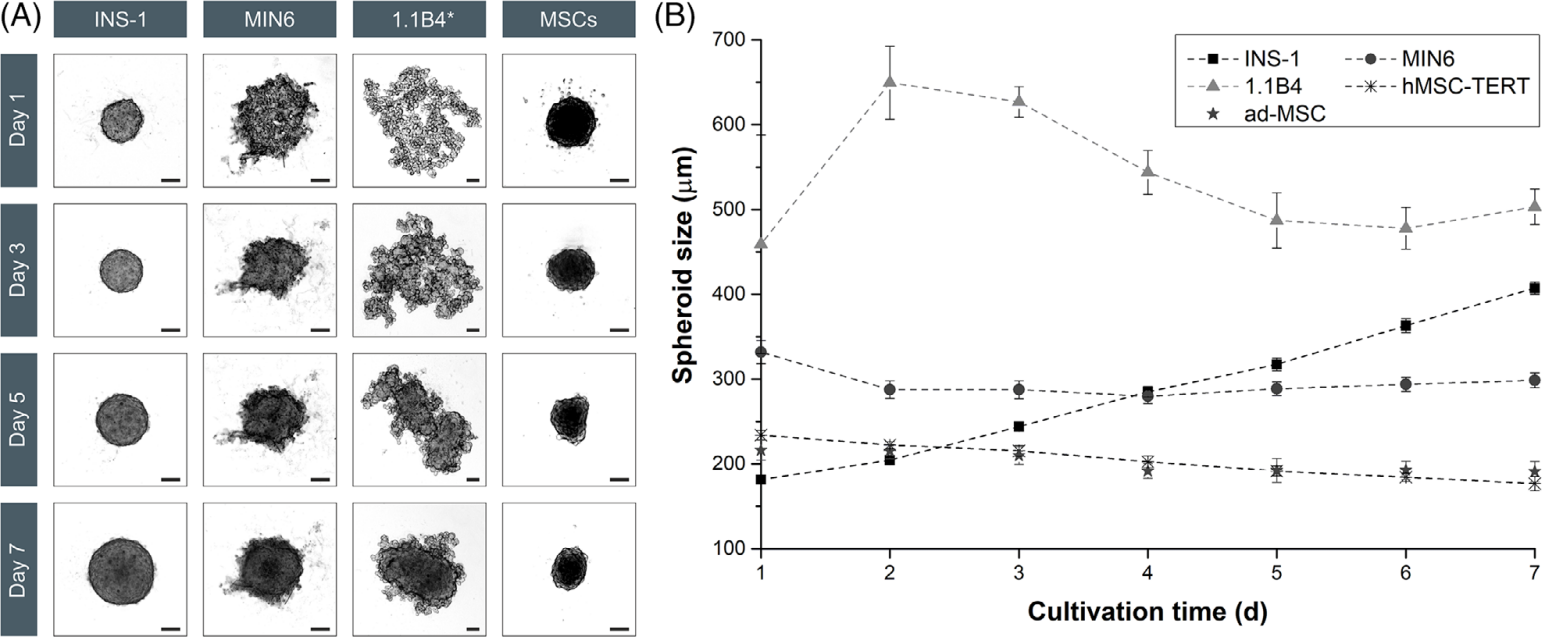
Images of the spheroids from static culture for morphological analysis and the determination of properties such as spheroid size and circularity (*n* = 12; data are means ± STDV). (A) Morphological differences can be seen during the aggregation of the different β‐cell lines within 7 days. The images of INS‐1, MIN6, and MSCs were reduced by 60%, whereas the 1.1B4 images were reduced by 73% (^*^). In both cases the scale bar represents 100 μm. (B) The growth kinetics of the spheroids reveal the differences between cell lines. The INS‐1 cells (squares) showed fast aggregation and a continuous increase in spheroid size, whereas the MIN6 cells (circles) needed 3 days to form stable spheroids before slow growth was observed. The 1.1B4 cells (triangles) aggregated slowly over 5–7 days, represented by a peak on day 2 followed by a decline in size until a stable spheroid was formed and a volume increase was observed. The hMSC‐TERTs (crosses) and ad‐MSCs (stars) showed no growth and the spheroid size declined over time. 1.1B4, a cell line formed by the electrofusion of primary human pancreatic islets and PANC‐1 cells; hMSC‐TERT, human mesenchymal stromal/stem cells immortalized with reverse transcriptase telomerase; INS‐1, rat insulinoma‐1 cell line; MIN6, mouse insulinoma‐6 cell line; MSC, mesenchymal stromal/stem cell; STDV, standard deviations

The INS‐1 cells formed stable spheroids (*Ø* = 195 ± 8 μm), clearly distinct from the surrounding culture medium after 1 day, whereas the MIN6 and 1.1B4 cells formed loose cell clusters with large gaps and irregular edges, especially 1.1B4 cells (Figure 1A). The MIN6 cells required ∼3 days to transform this loose structure into a stable spheroid (*Ø* = 288 ± 11 μm). The loose structure of 1.1B4 cells was characterized by the formation of smaller sub‐aggregates that assembled into spheroids (*Ø* = 503 ± 21 μm) after 7 days. Slow spheroid formation was previously reported for 1.1B4 cells, which needed 5 or 7 days to form spheroids with a diameter of 280 ± 60 μm [21] or 100–200 μm [22]. The aggregation and compaction of 1.1B4 spheroids were slower compared to INS‐1 and MIN6 spheroids (Figure 1A).

A second criterion for the quality of spheroid formation is circularity (C), which evaluates the degree of roundness. Within 24 h, the INS‐1 spheroids achieved a value of *C* = 0.93 ± 0.04, compared to *C* = 0.46 ± 0.06 for MIN6, and *C* = 0.51 ± 0.05 for 1.1B4. Over a period of 7 days, INS‐1 spheroids retained their smooth surface, whereas the surface of MIN6 and 1.1B4 spheroids was more heterogeneous (lower circularity), featuring single cells or small aggregates (Figure 1A). The circularity reflects the surface properties of the spheroids and indicates the aggregation behavior of the cells, meaning that higher *C* values correspond to faster aggregation.

The three β‐cell lines differed in their spheroid growth behavior. The INS‐1 spheroids grew continuously, increasing in diameter (Figure 1B). The rapid growth of INS‐1 spheroids has been reported elsewhere [23]. However, the growth of MIN6 spheroids stagnated until compaction was completed (day 4), and overall growth was therefore slow (Figure 1B). The fast and slow growth of the INS‐1 and MIN6 cells, respectively, in 2D (Supporting Information Table S1) was therefore recapitulated in 3D culture. The INS‐1 spheroids steadily increased in volume (*μ*_Vol_ = 0.327 ± 0.013 day^–1^; *t*_D,Vol_ = 2.12 ± 0.08 days), whereas MIN6 (*μ*_Vol_ = 0.069 ± 0.007 day^–1^; *t*_D,Vol_ = 10.0 ± 1.0 days) and 1.1B4 (*μ*_Vol_ = 0.044 ± 0.014 day^–1^; *t*_D,Vol_ = 16 ± 5 days) spheroids were slower, once aggregation was complete.

The size of the MIN6 and INS‐1 spheroids and their good or very good aggregation properties, respectively, agree with previous reports [3, 13, 23, 24], probably reflecting their origin from an insulinoma with strong cell–cell contacts [25]. The slow aggregation behavior of 1.1B4 cells [21, 22] may reflect the origin of this cell line as an electrofusion of primary adult β‐cells and the cell line PANC‐1, thus displaying bipartite characteristics. The morphology, growth, and the weak insulin secretion under 2D conditions already indicate that the properties of the epithelial cell line PANC‐1 are dominant. Furthermore, the PANC‐1 cell line is derived from an adenocarcinoma of ductal exocrine cells, thus the cells tend to form few cell layers [26], which could also explain the limited aggregation behavior of our spheroids. When comparing the characteristics of monolayers and 3D cultures (Supporting Information Figure S1), we can conclude that the tendency to form clusters in 2D also promotes the aggregation of β‐cells (INS‐1 and MIN6) in contrast to the epithelial characteristics of 1.1B4 cells.

**FIGURE 2:**
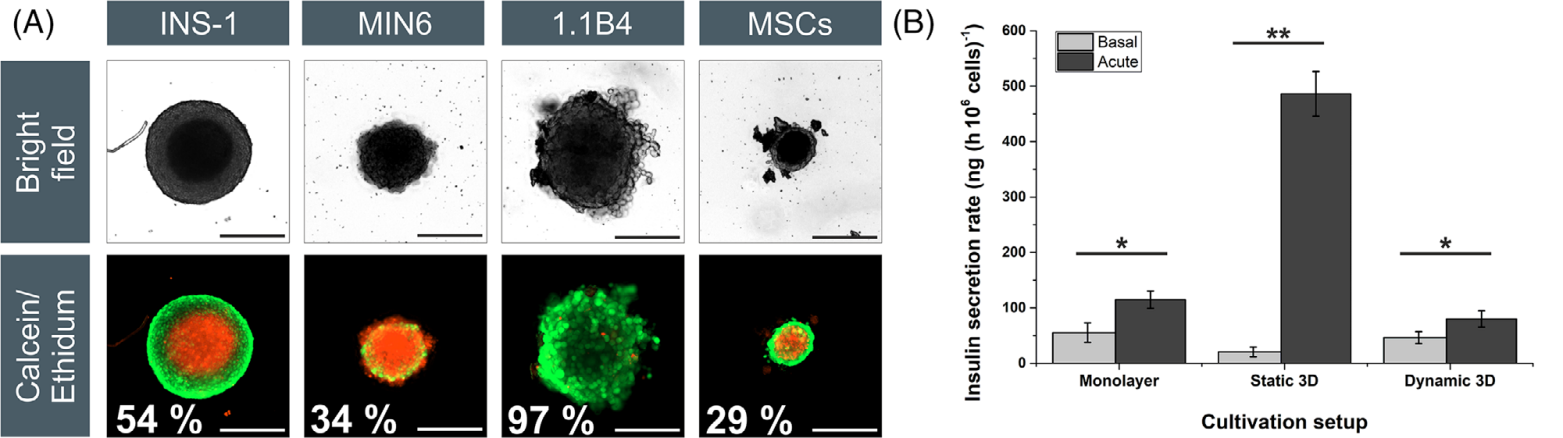
The viability of spheroids from static cultures determined by staining with calcein AM and ethidium after 7 days. (A) INS‐1 spheroids featured a dead core and a viable mantle, whereas MIN6 spheroids featured a heterogenous distribution of dead cells. The loose structure of the 1.1B4 spheroids promoted sufficient mass transfer resulting in a high viability. (B) Insulin secretion profiles of INS‐1 cells cultured as monolayers and spheroids cultured under static (96‐well plate) and dynamic (shaking flask) conditions (*n* = 3; data are means ± STDV; significance intervals are *p < 0.05, **p < 0.01, and ***p < 0.001). 1.1B4, a cell line formed by the electrofusion of primary human pancreatic islets and PANC‐1 cells; INS‐1, rat insulinoma‐1 cell line; MIN6, mouse insulinoma‐6 cell line; STDV, standard deviations

The viability of the spheroids was determined after 7 days in culture (Figure 2A). The INS‐1 spheroids showed a viability of 54% ± 25% and formed a distinct dead spheroid core as early as 24 h after seeding. This probably reflects the limited mass transfer of nutrients and/or the accumulation of metabolites. The diffusive distance between the outer layer of the INS‐1 spheroids and the dead core was 68 ± 5 μm (Supporting Information Figure S2). Although a diffusive limit of 150–200 μm in spheroids has been reported for oxygen [27], this is highly dependent on the culture medium, the compactness of the spheroids, the cultivation method, and the cell type. In particular, the U‐bottom plates we used and the level of the culture medium may have imparted a negative effect. Although an increase in the diffusive distance between the gas phase and the cells has a significant effect on the oxygen supply, a reduction in the spheroid surface area, due to the parabolic shape of the wells (which corresponds to the spheroid shape), also has a negative effect on mass transfer within the spheroids [28]. The INS‐1 spheroids had already reached a diameter of 195 μm after 24 h, thus exceeding the permissible diffusive barrier of 136 μm, which probably explains the death of the internal cells. The MIN6 spheroids showed a low viability of 34% ± 3%, in contrast to previous studies reporting viabilities of 95% [24], 73%–84% [29], or 63% [30]. The reason for this discrepancy is unclear, but may reflect differences in spheroid formation, culture conditions, and the method used to measure viability. Interestingly, the MIN6 spheroids showed dead cells distributed evenly within the spheroid, with living cells on the outer layers and within the core (Figure 2A). The loose structure of the 1.1B4 spheroids favored a diffusive mass transfer, resulting in a high viability of 97% ± 3%, in agreement with previous studies [22].

The functionality of the β‐cells was determined (Table 1). All β‐cell spheroids showed some level of glucose‐dependent insulin secretion. The 1.1B4 spheroids, in contrast to the monolayer cultures, showed a weak insulin secretion. The MIN6 spheroids secreted the highest amount of insulin, as was the case under 2D conditions, followed by the INS‐1 spheroids, and showed a significant response to the higher glucose concentration (**p < 0.05).

**TABLE 1:** The insulin profiles of β‐cell spheroids in static culture was measured by GSIS (*n* = 3; error = STDV)

	Insulin secretion profile			
Cell type	Basal	Acute	SI [–]	Reference
1.1B4	0.09 ± 0.07 pg (spheroid h)^–1^ 0.019 ± 0.015 ng mL^–1^	0.4 ± 0.6 pg (spheroid h)^–1^ 0.08 ± 0.11 ng mL^–1^	1 ± 2	This study
	0.18 ng mL^–1^	0.35 ng mL^–1^	2	[22]
INS‐1	3.8 ± 1.7 pg (spheroid h)^–1^ 0.8 ± 0.3 ng mL^–1^	91 ± 8 pg (spheroid h)^–1^ 18.1 ± 1.5 ng mL^–1^	23.5 ± 0.5	This study
	20 ng mL^–1^	40 ng mL^–1^	2	[23]
MIN6	13 ± 3 pg (spheroid h)^–1^ 7.4 ± 0.3 ng mL^–1^	200 ± 12 pg (spheroid h)^–1^ 40 ± 2 ng mL^–1^	5.36 ± 0.10	This study
	2.5 ng mL^–1^	6.25 ng mL^–1^	2.5	[61]

It is difficult to compare insulin secretion rates with other publications because the culture conditions and methods vary greatly, and insulin secretion is not normalized per spheroid or cell. Nevertheless, we confirmed a comparable trend of improved functionality due to 3D cultivation. 1.1B4, a cell line formed by the electrofusion of primary human pancreatic islets and PANC‐1 cells; INS‐1, rat insulinoma‐1 cell line; MIN6, mouse insulinoma‐6 cell line; SI, insulin stimulation index; STDV, standard deviations.

To improve the comparability between 2D and 3D cultures, a conversion factor [31, 32] was introduced to estimate the cell number in each spheroid. The conversion factor for INS‐1 was 15 × 10^–5^ ± 1.8 × 10^–5^ cells μm^3^, and is based on the assumption of a fast and compact spheroid formation (as is the case for this cell line). The conversion factor also accounts for the cell viability, resulting in a basal insulin secretion rate of 20.7 ± 8.8 ng (10^6^ cells h)^–1^ and a significant (**p < 0.01) increase in the acute secretion rate of 486.2 ± 40 ng (10^6^ cells h)^–1^ for INS‐1 spheroids (Figure 2B). The conversion factor allowed us to compare the 3D secretion profile with the existing data for 2D cultures. The insulin secretion per cell was significantly (***p < 0.001) higher (2D = 47 [basal] to 95 [acute] ng (10^6^ cells h)^–1^), and the insulin stimulation index (SI) increased 11‐fold. The lower continuous basal insulin secretion with a simultaneous increase in the response to a higher glucose concentration suggests that the functionality of INS‐1 spheroids improved, which in turn may reflect the in vivo‐like 3D environment [33].

#### 3.1.2 Dynamic spheroid formation

We initially used simple systems to assess the general aggregation behavior of β‐cell lines under dynamic conditions to establish whether a dynamic cultivation can increase the spheroid viability. We first investigated shaking 12‐well plates and spinner flasks because these systems allow simple and quick preliminary studies of dynamic aggregation. However, they result in heterogeneous mixing [34]. The shaking 12‐well plates allowed the aggregation of all β‐cell lines after only 24 h (Supporting Information Figure S3). The INS‐1 cells formed large, compact structures up to 2 cm in diameter with a smooth surface. These irregular structures may indicate the very rapid aggregation of individual cells into spheroids, which subsequently aggregated into these even larger structures. MIN6 cells formed irregular aggregates with a heterogeneous surface (*Ø* = 231 ± 91 μm), whereas the 1.1B4 aggregates were much more spherical, but had a loose structure and a heterogeneous surface (*Ø* = 381 ± 94 μm).

The scale‐up of the dynamic spheroid formation in spinner flasks supported the results presented above. For the first 2 days, the viability of the spheroids was ∼100% (except for some single cells), indicating a gentle aggregation. The dynamic formation of much larger aggregates, compared to static conditions, reflected the greater viability of the cells and confirmed the more efficient mass transfer. In other studies, MIN6 spheroids in spinner flasks formed aggregates of 100–400 μm with a greater viability than in static cultures [35]. Furthermore, MIN6 spheroid cultures showed lower levels of a lactate dehydrogenase and caspase activity, and lower amounts of fragmented DNA (measured using a TUNEL assay) [35]. As stated above, mixing or power input is heterogeneous in multi‐well plates and spinner flasks, reflecting the lack of isotropic turbulence. This promotes the uncontrolled aggregation of cells or spheroids (especially INS‐1 cells) with a broad size distribution. Shaking cultures of the β‐cell line RIN‐5F gave comparable results, also with a broad spheroid size distribution [36].

**FIGURE 3:**
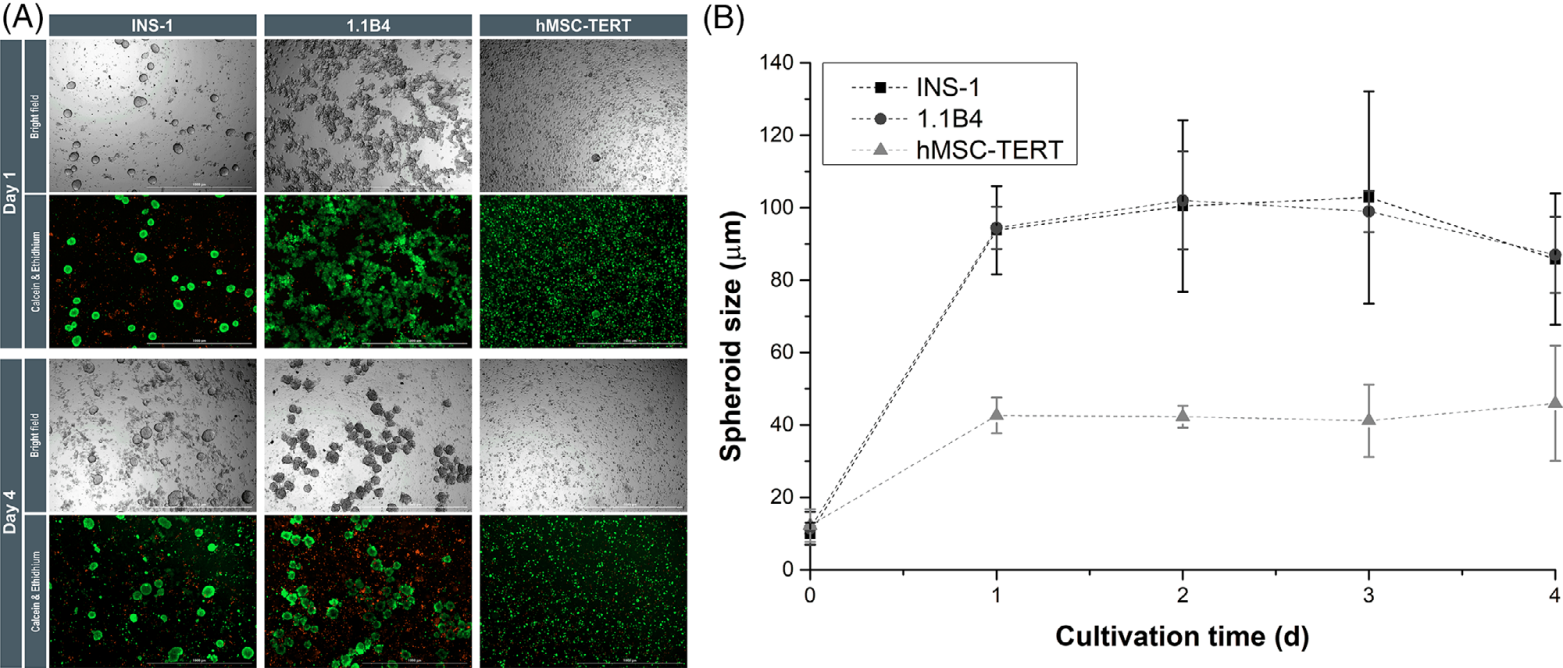
Spheroid viability and growth. (A) Bright‐field images and viability of monospheroids generated in shaking flasks after 1 and 4 days. INS‐1 cells (left) produced uniform spheroids, whereas 1.1B4 cells (second column) needed 2 days to form stable spheroids. The hMSC‐TERTs (third column) produced small spheroids that decreased in size. Scale bar = 1000 μm. (B) Spheroid growth in shaking flasks under dynamic conditions. The INS‐1 (black squares) and 1.B4 (black circles) cells showed similar behavior, forming spheroids (*d*_32_ = 94 μm) after 1 day. The hMSC‐TERTs (gray triangle) formed smaller spheroids (*d*_32_ = 43 μm). In all cases, the shaking flasks restricted the spheroid size (*n* = 3; data are means ± STDV). 1.1B4, a cell line formed by the electrofusion of primary human pancreatic islets and PANC‐1 cells; hMSC‐TERT, human mesenchymal stromal/stem cells immortalized with reverse transcriptase telomerase; INS‐1, rat insulinoma‐1 cell line; STDV, standard deviations

Accordingly, we used baffled shaking flasks to achieve a more homogenous energy distribution. Using established models by [37, 38, 39, 40] to assess the power consumption within the shaking flaks, we obtained a fully turbulent fluid dynamic and Reynolds number Re > 10.000 at a shaking speed of 100 rpm. We analyzed the dynamic spheroid formation in the two β‐cell lines with the extreme behaviors under static conditions (INS‐1 and 1B4, with the best and worst performance, respectively). INS‐1 cells benefited from the turbulent flow in the shaking flasks and formed smaller spheroids (*d*_32_ = 94 ± 12 μm; *C* = 0.68 ± 0.02; Figure 3). This suggests that the hydrodynamic forces in the 12‐well plates were too weak, so that most of the spheroids aggregated into larger structures (Supporting Information Figure S3). The time span of the INS‐1 spheroid formation was comparable in static and dynamic culture (both 24 h). Under dynamic conditions, the circularity of the spheroids was 26% lower, reflecting the different mechanisms of static and dynamic aggregation. Under dynamic conditions, the suspended cells must collide and establish cell–cell contacts, so aggregation proceeds until all suspended cells are aggregated, or the hydrodynamic forces of the fluid prevent further cell–cell connections. Which of the two principles prevails, depends on the hydrodynamic conditions and the system, as previously discussed in detail [41]. The 1.1B4 cells formed stable spheroids after 2 days, whereas 5–7 days were required under static conditions. After 1 day, the 1.1B4 spheroids achieved the following properties: *d*_32_ = 94 ± 6 μm and *C* = 0.56 ± 0.04 (Figure 3).

Under static conditions and non‐turbulent dynamic conditions, we only observed the continuous growth of INS‐1 spheroids. In the shaking flasks (Figure 3B), under turbulent conditions, the INS‐1 and 1.1B4 showed a volume increase of *μ*_Vol_ = 0.14 ± 0.04 day^–1^ (*t*_D,Vol_ = 5.0 ± 1.4 day) and *μ*_Vol_ = 0.07 ± 0.09 day^–1^ (*t*_D,Vol_ = 10 ± 13 days), respectively, between the completed spheroid formation (1 day) and day 3, until the spheroids decreased on day 4. The change from static or non‐turbulent dynamic to turbulent dynamic conditions limited the volume increase of the spheroids. This probably reflects continuous stress‐induced shearing off of cells from the spheroid due to surface erosion [41]. The regrowth of cells on the spheroid surface is restricted, so the number of single cells or small aggregates (*d* < 20 μm) increased in the supernatant. On day 1, dead INS‐1 cells in the supernatant represented 10% ± 2% and increased to 74% ± 3% on day 4. The 1.1B4 cells showed a similar behavior (4.7% ± 0.3% dead cells on day 1, increasing to 33% ± 14% on day 4). This reflects the isotropic turbulence within the shaking flasks, combined with shear forces eroding the spheroid surface, leading to the anticipated size restriction. However, the viability of spheroids for all cell types remained at ∼100%, except for some single internal cells (Figure 3A). Dynamic spheroid cultures have previously been described for β‐cells/islet cells [35, 42], tumor cell lines [43], and iPSCs [44], but most were in non‐baffled spinner flasks with a low power input and no turbulent flow pattern, so they cannot be compared directly with our system.

In terms of spheroid formation (rapidity, circularity, and size distribution), the INS‐1 cells performed the best and were therefore used for subsequent functionality testing, especially given that 1.1B4 cells secrete very low amounts of insulin. Under dynamic conditions, the INS‐1 spheroids showed a basal glucose‐dependent (*p < 0.05) insulin secretion of 6 ± 3 pg insulin (spheroid h)^–1^ and an acute secretion of 12.2 ± 0.4 pg insulin (spheroid h)^–1^, resulting in an SI of 2.4 ± 1.0. Using our established conversion factor, we found that the insulin profile of our dynamic spheroids was similar to that of the 2D cultures (Figure 2B). Dynamic INS‐1 spheroids secreted insulin at a basal rate of 46 ± 11 ng (10^6^ cells h)^–1^ and at an acute rate of 80 ± 15 ng (10^6^ cells h)^–1^, similar to previous studies [23]. These results clearly indicate the increased mass transfer in shaking flasks, confirming that we were able to establish a scalable dynamic spheroid formation as a basis for the large‐scale production of viable and functional spheroids in a defined size range. This achievement must be transferred to primary β‐cells or iPSCs.

### 3.2 Formation of spheroids from MSCs

#### 3.2.1 Static spheroid formation

The hMSC‐TERT (*Ø* = 243 ± 6 μm) and ad‐MSC (*Ø* = 216 ± 12 μm) cultures formed stable spheroids, clearly distinct from the surrounding culture medium after 1 day (*C* = 0.72 ± 0.04 and 0.68 ± 0.09 for hMSC‐TERTs and ad‐MSCs, respectively). We observed no differences between primary ad‐MSCs and the hMSC‐TERT cell line, and our data agreed with earlier reports [45]. The 2D cultures featured a homogenous MSC distribution without cluster formation (like 1.1B4 cells), but hMSC‐TERT and ad‐MSC cultures nevertheless showed a strong tendency to form spheroids within 24 h. This behavior was not anticipated based on the 2D behavior – for example, 2D MSCs strictly need a growth surface to avoid anoikis. Furthermore, the rapid MSC growth observed as monolayer culture (hMSC‐TERT *μ*_2D_ = 0.70 ± 0.03 day^–1^; ad‐MSC *μ*_2D_ = 0.73 ± 0.06 day^–1^) was not replicated in form of spheroids, and the volume of the MSC spheroids decreased (*μ*_Vol_ = –0.06 ± 0.10 day^–1^) (Figure 1A and B). MSCs in 3D cultures undergo a continuous rearrangement, and lose up to 75% of their volume, resulting in a compact structure with small round cells in the core, and elongated cells around the mantle [45, 46, 47, 48].

The hMSC‐TERT and ad‐MSC static 3D cultures also resulted in the formation of dead spheroid cores, and the viability fell to 29% ± 13% (Figure 2A), supporting earlier results [49]. The diffusive distance was 11 ± 2 μm for MSC spheroids, so the mean diameter of 194 μm exceeded the maximum size by far, and thus explains the low viability. The formation of a dead spheroid core could also explain the volume reduction.

#### 3.2.2 Dynamic spheroid formation

We were unable to find any differences between the primary ad‐MSCs and hMSC‐TERTs, so we only used the hMSC‐TERT line for a dynamic spheroid formation, as discussed above for the β‐cell lines. After 24 h in the 12‐well plates, the hMSC‐TERTs formed smaller spheroids (*Ø* = 84 ± 28 μm) than the β‐cell lines (Supporting Information Figure S3), but we also observed MSCs adhering to the surface. The hMSC‐TERTs in spinner flasks behaved in a similar manner. Live/dead staining revealed that the spheroids were ∼100% viable. The adherence of cells to the surface of the plates confirmed that MSCs are highly dependent on a growth surface, but also revealed the insufficient power input of this system. In shaking flasks, the hMSC‐TERTs formed 50% smaller spheroids (*d*_32_ = 43 ± 5 μm; *C* = 0.54 ± 0.03; Figure 3B). After the spheroid formation, the volume of the hMSC‐TERTs declined by *μ*_Vol_ = –0.053 ± 0.016 day^–1^. Stagnation and volume reduction (rearrangement and densification) thus affected MSC spheroids under static and dynamic conditions. In the latter case, the volume reduction cannot be explained by cell death because the spheroid viability was ∼100% (except some individual cells). The number of single cells suspended in the supernatant increased from 10% ± 2% on day 1 to 33% ± 2% on day 4, reflecting the surface erosion discussed above.

### 3.3 Coculture of β‐cells and MSCs

Native human islets consist of a majority of centrally‐located β‐cells surrounded by a smaller number of α‐cells, δ‐cells, and PP‐cells [50, 51]. MSCs confer benefits on islets in coculture experiments [14, 52], but this depends on the type of β‐cell and MSC. We used the static spheroid formation to investigate the interactions between MSCs and β‐cells in a 3D microenvironment. We investigated how MSCs affect the aggregation behavior, growth, viability, and insulin secretion, and determined the optimal β‐cell to MSC ratio, which is particularly important for scaling‐up the process.

**FIGURE 4:**
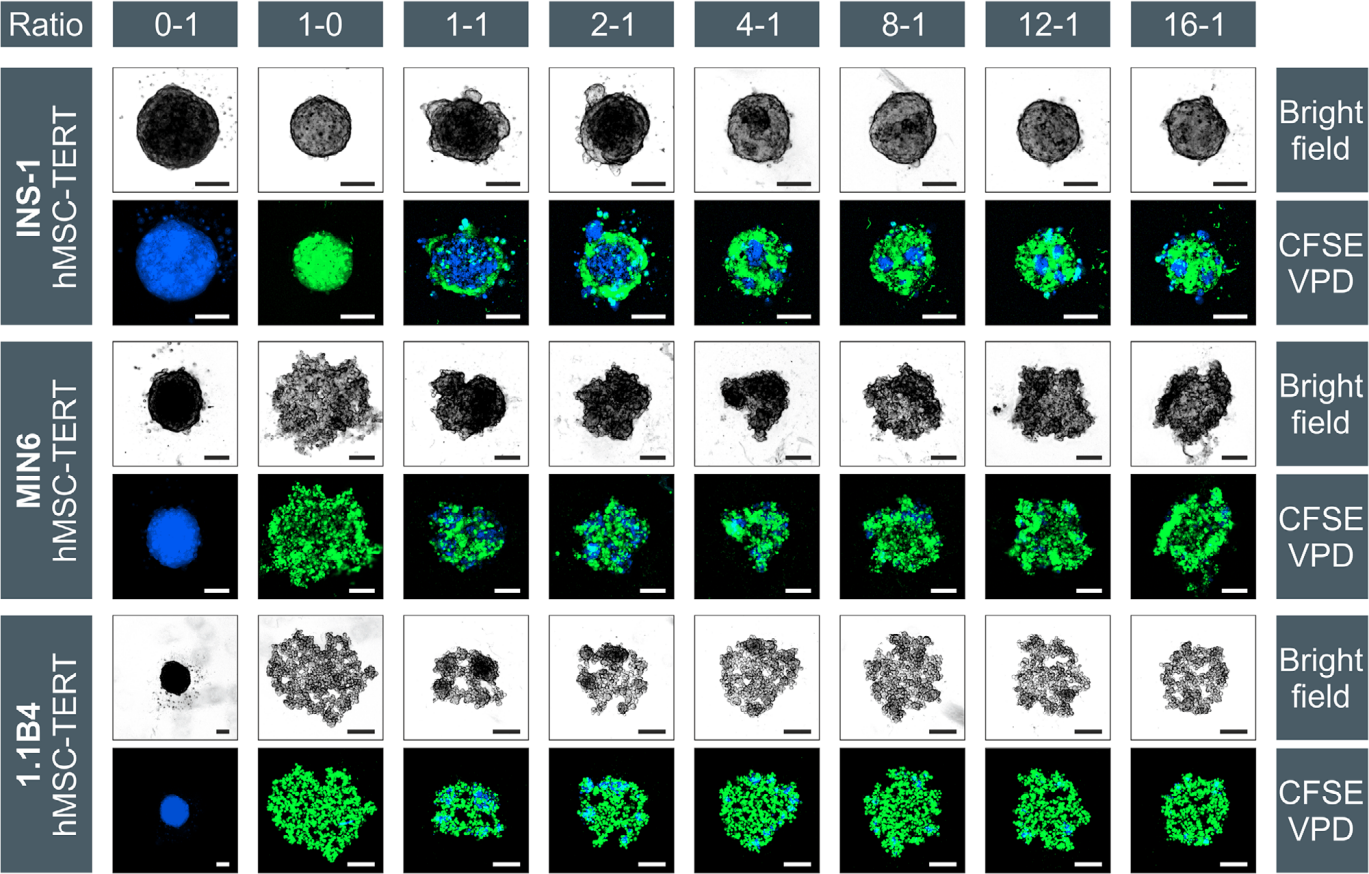
Aggregation of INS‐1 (upper row), MIN6 (middle row) and 1.1B4 (lower row) cells with hMSC‐TERTs at different ratios after 24 h. MSCs were stained blue (VPD) and β‐cells were stained with the green dye CFSE. Starting with monospheroids in the first (MSCs, blue) and second (β‐cells, green) columns, the cell ratios increase from left to right. Due to different scaling of the images, the MSC spheroids seem to have a different size in each setup, but the seeding density was always 1000 cells per well. In all cases the scale bar represents 100 μm. 1.1B4, a cell line formed by the electrofusion of primary human pancreatic islets and PANC‐1 cells; CFSE, 5‐(and 6)‐carboxyfluorescein diacetate, succinimidyl ester; hMSC‐TERT, human mesenchymal stromal/stem cells immortalized with reverse transcriptase telomerase; INS‐1, rat insulinoma‐1 cell line; MIN6, mouse insulinoma‐6 cell line; MSC, mesenchymal stromal/stem cell

Long‐term cell labeling allowed us to follow the spatial distribution of cocultured β‐cells and MSCs (Figure 4). Heterospheroids were formed by all three β‐cell lines that were cocultured with hMSC‐TERTs, but the cells always remained segregated, rather than achieving a homogeneous distribution. The same phenomenon was observed for INS‐1 cells, cocultured with ad‐MSCs. After the spheroid formation at β‐cell/MSC ratios of 1:1 to 4:1, the MSCs formed the spheroid core with β‐cells as the mantle. Reducing the proportion of MSCs (β‐cell/MSC ratios of 8:1 and 16:1) resulted in the clustering of MSCs within the spheroids. This was particularly evident for the rapidly aggregating cell line INS‐1, and at later time points, for MIN6 and 1.1B4. A similar core–shell configuration was previously reported for re‐aggregated human islet cells [53]. Although a direct coculture had no effect on the aggregation speed of INS‐1 heterospheroids, their size increased significantly at β‐cell/MSC ratios of 1:1 to 4:1 (***p < 0.001), reflecting the larger size of the hMSC‐TERTs, compared to INS‐1 cells. The aggregation of MIN6 and 1.1B4 cells was accelerated in the presence of MSCs, with MIN6 heterospheroids appearing after only 1 day, and 1.1B4 spheroids forming after only 3–4 days instead of the typical 5–7 days. Accordingly, all heterospheroids were smaller on day 1 (*Ø* = 255 ± 7 μm) than the corresponding monospheroids of MIN6 (*Ø* = 332 ± 14 μm; ***p < 0.001). The difference was particularly evident for the 1.1B4 heterospheroids, whose diameter (*Ø* = 498 ± 38 μm) was 35% lower than that of the monospheroids (*Ø* = 649 ± 43 μm; ***p < 0.001). The rapid aggregation of hMSC‐TERTs may explain the accelerated aggregation and densification observed in cocultures, acting as a nucleus for the spheroid formation by favoring the aggregation of the MIN6 and 1.1B4 cells. Significant differences in circularity were observed between the INS‐1 monospheroids and heterospheroids. The circularity values decreased from 0.97 to 0.69 at β‐cell/MSC ratios 1:1 to 4:1 (**p < 0.01), but increased again with decreasing amounts of MSCs, probably due to the continuous rearrangement and compaction of the MSCs [23, 45–48]. Because the MIN6 and 1.1B4 cells formed spheroids that were irregular, compared to INS‐1 cells and MSCs (single cells and small aggregates on the surface, respectively), no significant change was detected with respect to the circularity of the heterospheroids.

The MSCs had no effect on the growth behavior of INS‐1 spheroids, resulting in a similar growth curve as in Figure 1. The mean volume‐related growth rate was *μ*_Vol_ = 0.309 ± 0.015 day^–1^ (*t*_D,Vol_ = 2.25 ± 0.12 days). Relative to the MIN6 monospheroids (*μ*_Vol_ = 0.069 ± 0.007 day^–1^), the growth rate of the 1:1 β‐cell/MSC ratio coculture initially decreased (*μ*_Vol_ = 0.008 ± 0.011 day^–1^), but increased again as the proportion of MSCs decreased (16:1, *μ*_Vol_ = 0.129 ± 0.015 day^–1^). In contrast, the growth rate for 1.1B4 cells increased relative to the monospheroids (*μ*_Vol_ = 0.044 ± 0.014 day^–1^). Here, β‐cell/MSC ratios of 1:1 (*μ*_Vol_ = 0.102 ± 0.009 day^–1^) and 16:1 (*μ*_Vol_ = 0.092 ± 0.034 day^–1^) achieved the highest growth rates, whereas ratios of 2:1 to 12:1 resulted in a slower growth (*μ*_Vol_ = 0.066 day^–1^). The increase in growth may reflect the growth‐promoting properties of the MSCs, but in our opinion they are probably connected to the accelerated aggregation and densification of the spheroids (due to the MSCs) and the related analytics. The diameter of the MIN6 and 1.1B4 heterospheroids decreased due to compression, leaving less free space within the spheroids, in contrast to the looser structures of the monospheroids. But this led to a radial cell expansion, which explains the increase in volume‐related growth rates.

The spontaneous segregation of cocultured β‐cells and MSCs may reflect the different species of the cells (human, rat, and mouse), or the different origins. However, the coculture of hMSC‐TERTs and 1.1B4 cells (both human) also resulted in segregation. The complete separation of primary islet cells and MSCs has also been described [15]. Initially homogeneous heterospheroids containing both cell types, divided into separate monospheroids within 3 d. Similar results were reported for the β‐cell line EndoC‐βH3 and HUVECs [51]. This phenomenon is strongly cell‐type‐dependent. The 3D coculture of MIN6 and other cell types, such as C2C12, 3T3L1, and Colon26 preferentially resulted in the MIN6 cells segregating to the mantle of the spheroids, whereas the coculture with HepG2 and MAEC cells resulted in a homogeneous cell distribution [54]. We never observed MIN6 cells in the core of our spheroids, which may be due to the FAK signaling‐dependent migration of MSCs to the core, explaining the formation of β‐cell mantles [54]. Two other theories could explain these results: the differential adhesion hypothesis (DAH) [55], and the differential interfacial tension hypothesis (DITH) [56]. The DAH describes, how cell aggregates replace weak adhesive bonds with strong ones, forcing irregular structures into a spherical morphology, and reducing the surface tension of cell aggregates [55, 57]. The surface tension of hMSC‐TERTs is increased by the expression of N‐cadherins on the surface, such that INS‐1 cells (with fewer N‐cadherins and thus lower surface tension) tend to form a mantle. This is supported by the formation of heterospheroids with a β‐cell core, due to high levels of E‐cadherin, and a HUVEC mantle due to the absence of E‐cadherins [51]. The DITH considers the thermodynamic processes between the interfaces of the cells (δ_cell‐cell_) and the cells with the surrounding culture medium (δ_cell‐medium_). The formation of the MSC core and β‐cell mantle would imply that the homospecific MSC–MSC connection (δ_MSC‐MSC_) is thermodynamically more favorable than the heterospecific interface (δ_β‐cell‐MSC_), but the latter is better than the medium–MSC interface (δ_MSC‐medium_), which can be expressed as δ_MSC‐MSC_ < δ_β‐cell‐MSC_ < δ_MSC‐medium_ [56]. Differences in cadherin expression and interfacial tensions between individual cells in our spheroids should be investigated, to determine if these hypotheses can explain our results.

**FIGURE 5:**
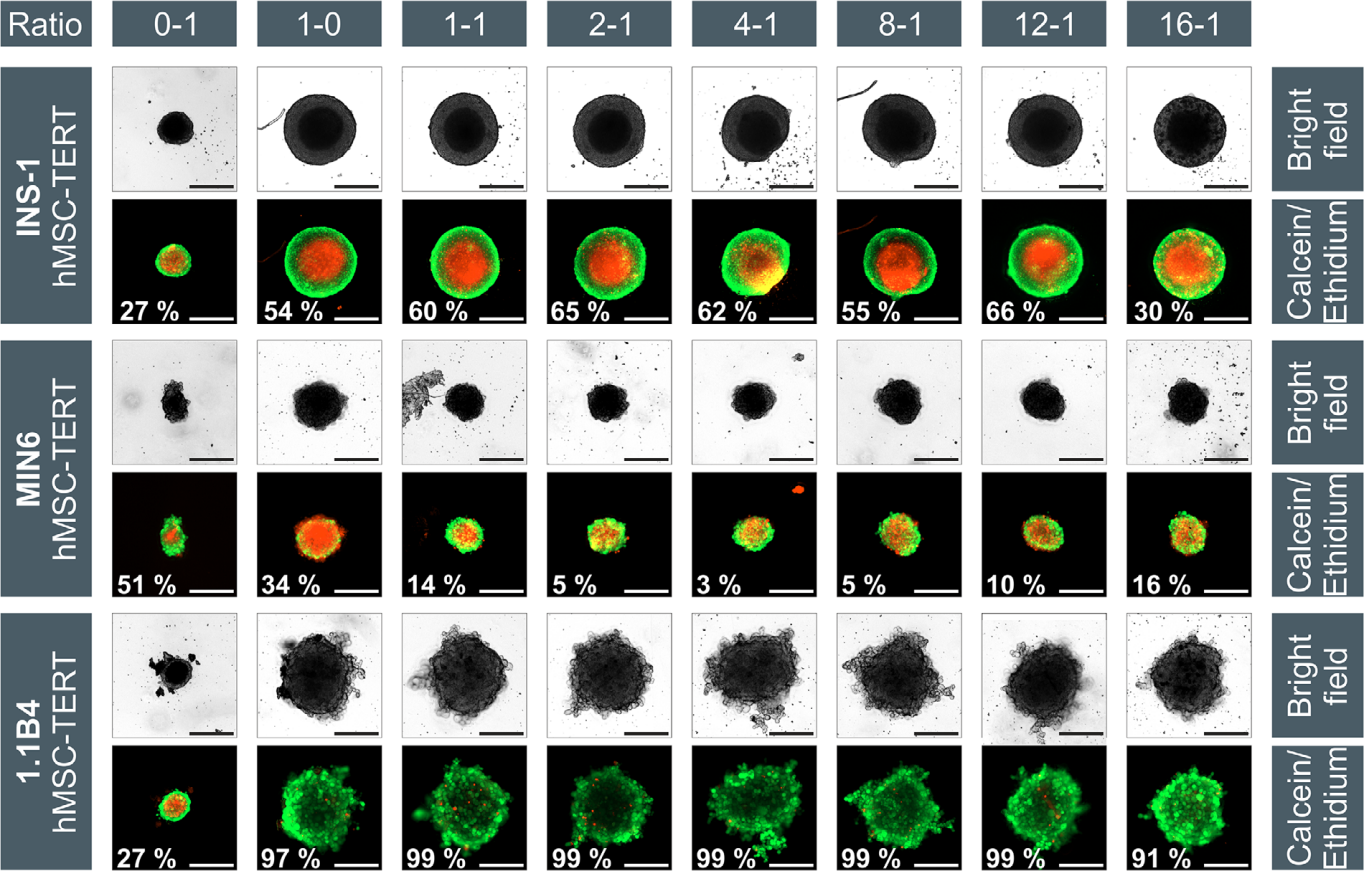
Bright‐field and viability images (at day 7) of monospheroids (0–1 = MSC only; 1–0 = β‐cell only) and heterospheroids INS‐1 (upper row), MIN6 (middle row), and 1.1B4 (lower row) cocultured with hMSC‐TERTs at different cell ratios. The stated viabilities of the spheroids were assessed by measuring the red (core) and green (whole spheroid) diameter and the resulting volume to describe the real “3D viability,” but the displayed images only represent two dimensions of the spheroids, which could provide a deceptive impression. Scale bar = 100 μm. 1.1B4, a cell line formed by the electrofusion of primary human pancreatic islets and PANC‐1 cells; hMSC‐TERT, human mesenchymal stromal/stem cells immortalized with reverse transcriptase telomerase; INS‐1, rat insulinoma‐1 cell line; MIN6, mouse insulinoma‐6 cell line; MSC, mesenchymal stromal/stem cell

To determine the influence of MSCs on β‐cells, we measured spheroid viability (Figure 5) and functionality after 7 days. The viability of the heterospheroids was impaired by a limited mass transfer, as already shown for the monospheroids. The average viability of the INS‐1/MSC spheroids was 62% ± 5%, slightly higher than the 54% ± 25% viability of the INS‐1 monospheroids. In contrast, the average viability of the MIN6/MSC spheroids was 9% ± 5%, significantly (**p < 0.01) lower than the 34% ± 3% viability of the MIN6 monospheroids. Although the MIN6 cells formed the outer region of the spheroids, the loss of viability may reflect the 10‐fold higher substrate consumption rates of the hMSC‐TERTs, reducing the vital zone to ∼10 μm, which is just as small as that in the MSC monospheroids. The 1.1B4 spheroids achieved a high viability of 98% ± 2%. Although the MSCs accelerated the aggregation and densification, the structure of the 1.1B4 spheroids was still loose. There were no significant differences in viability between the 1.1B4 monospheroids and heterospheroids.

Given that the mass transfer had a strong effect on the static spheroid cultures, the functional analysis was suboptimal. We observed a very low level of insulin secretion from the 1.1B4 heterospheroids, but this was similar to the monospheroids and was highly error‐prone, so it was not considered further. The insulin profile of the MIN6 heterospheroids reflected the trends observed in the viability analysis. The monospheroids secreted the most insulin: 13 pg (spheroid h)^–1^ basal to 200 pg (spheroid h)^–1^ acute, SI = 5. The cocultured spheroids secreted an average of 27 pg (spheroid h)^–1^ basal and 50 pg (spheroid h)^–1^ acute, SI = 2. We again normalized the insulin secretion of the heterospheroids to the live cell number, using the conversion factor. Compared to the monospheroids, the coculture reduced the insulin secretion by 41%. Only spheroids with an INS‐1/MSC ratio of 2:1 showed an improvement (220%), corresponding to a basal secretion rate of 0.05 ng (10^6^ cells h)^–1^ and acute secretion rate 1.56 ng (10^6^ cells h)^–1^. Hybrid spheroids of islet cells and hepatocytes with different cell ratios resulted in comparable insulin release profiles for monospheroids and heterospheroids containing up to 25% hepatocytes [58]. Because islets and hepatocytes share a common progenitor, islet functions can improve through the transdifferentiation of hepatocytes to insulin‐producing cells. Furthermore, coculturing helps to improve the quality of primary islets, which are impaired by a limited mass transfer after a stressful isolation and the lack of vascularization. To investigate the impact of MSCs on β‐cell lines in terms of viability and functionality, a different experimental setup should be considered, which enhances mass transfer and impairs the robust β‐cell lines with STZ or ALX to amplify any effect of the MSCs [59, 60].

**FIGURE 6:**
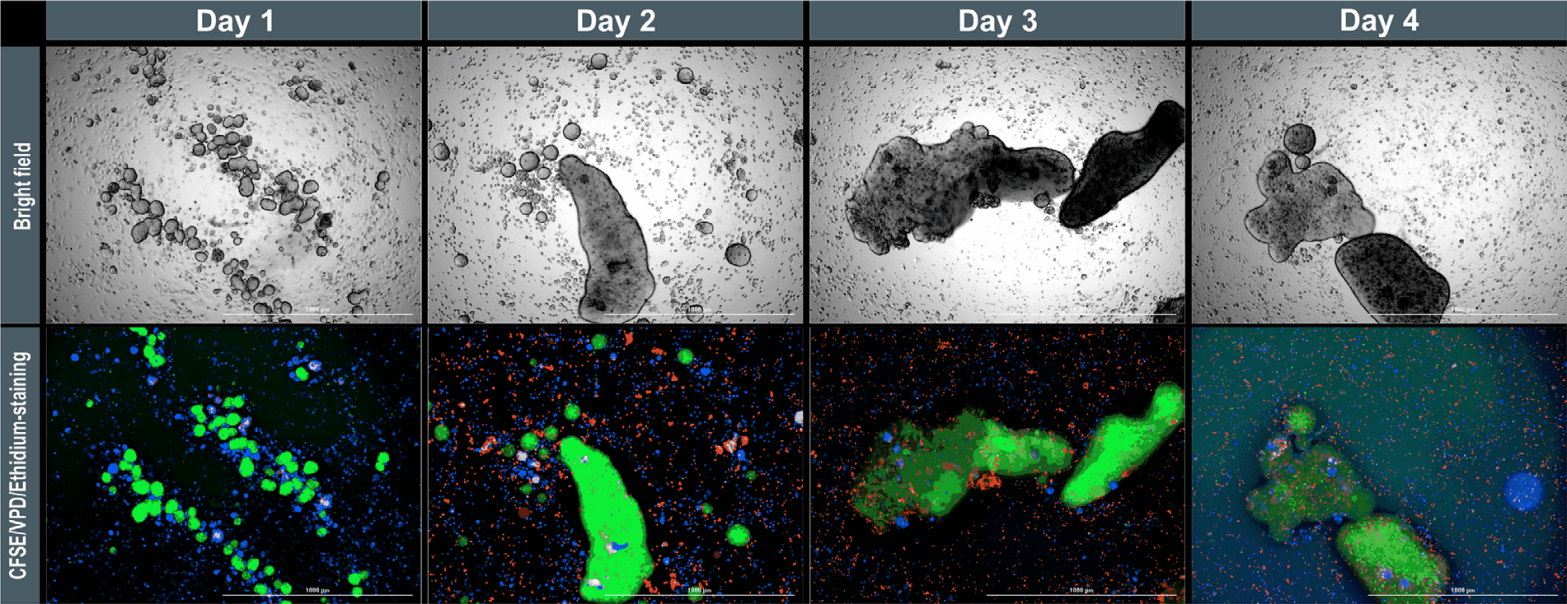
Coculture of INS‐1 cells and hMSC‐TERTs in shaking flasks with a 1:1 ratio. INS‐1 cells were stained with CFSE (green), hMSC‐TERTs with VPD (blue), and dead cells with ethidium (red). The long‐term dyes are diluted by cell division, so the images were overexposed from day 3 onwards to distinguish the cells. Scale bar = 1000 μm. CFSE, 5‐(and 6)‐carboxyfluorescein diacetate, succinimidyl ester; hMSC‐TERT, human mesenchymal stromal/stem cells immortalized with reverse transcriptase telomerase; INS‐1, rat insulinoma‐1 cell line

To produce highly viable heterospheroids, we established dynamic cultivation conditions in shaking flasks, and investigated the influence of MSCs on the β‐cells in terms of growth, viability, and functionality. Initial preliminary studies with the 1.1B4 cells and hMSC‐TERTs in spinner flasks resulted in the formation of large structures, presumably arising from the aggregation of the initial spheroids. In shaking flasks, staining with long‐term dyes helped to distinguish between the INS‐1 cells and hMSC‐TERTs (Figure 6), revealing the separation of each cell type into independent spheroids. This underscores the results from static cocultures, in which the spheroids featured MSC cores with a mantle of β‐cells, indicating that the separation of MSCs and β‐cells continued under dynamic conditions. We did not observe any changes in growth behavior, with both spheroid types showing similar *d*_32_ and *μ*_Vol_ values, compared to the monocultures in shaking flasks (Figure 3B). However, the aggregation of INS‐1 spheroids to form larger structures up to several millimeters in diameter was observed from day 2, whereas there was no further growth of hMSC‐TERT spheroids. Despite the size of the INS‐1 aggregates, the spheroids’ viability remained at ∼100% over the entire course of cultivation. Considering the DAH and DITH, the equilibrium seems to shift towards INS‐1 cells during the coculture period, perhaps due to the secretion of proteins from the MSCs in extracellular vesicles, and the subsequent adhesion of these proteins to the INS‐1 cell membrane. Alternatively, this may reflect the modification of the INS‐1 membrane secretome, due to a coculture with MSCs. Another indication of the changing membrane surface is the aggregation of the initial INS‐1 spheroids, which should be prevented by hydrodynamic forces. This change in aggregate strength should be investigated in more detail.

## 4 CONCLUDING REMARKS

We investigated spheroid formation by three different β‐cell lines and two types of MSCs under static conditions in a 96‐well format. This was useful to establish the analytics, and to characterize the aggregation behavior under different conditions. However, the viability of the spheroids in the static cultures was low, especially when cultivated for longer than 24 h. We showed that all three β‐cell lines were able to form β‐cell/MSC heterospheroids, but the two cell types did not mix completely, and instead segregated into different zones. Moreover, the β‐cell/MSC coculture did not improve viability under static conditions because this is predominantly influenced by mass transfer limitations. Under dynamic conditions, spheroids, representing all cell types, were ∼100% viable, and the β‐cell spheroids achieved a glucose‐dependent insulin secretion. We therefore recommend dynamic systems for screening, and for the investigation of cocultures. However, our work on dynamic coculturing has only just started, and further experiments are required to overcome the separation of cells under these conditions into separate monospheroids. Several approaches may favor the recovery of heterospheroids under dynamic conditions, including the modulation of the cadherin expression to promote cell mixing, and more extensive cell–cell contacts. Moreover, the experiments must be carried out with human islet cells or other model cells (e.g., EndoC‐βH1 cells) that bear a closer resemblance to native human β‐cells.

## NOMENCLATURE

**Table:** 

*C*	[–]	Circularity of the spheroids
*d*_32_	[μm]	Sauter diameter
SI	[–]	Insulin stimulation index
*t*_D,Vol_	[day]	Minimal time for doubling of volume
Re	[–]	Reynolds number
*V*_red_	[μm^3^]	Volume of dead cells
*V*_total_	[μm^3^]	Total volume of (viable + dead) cells
*μ*_2D_	[day^–1^]	Growth rate of cells cultured as a monolayer
*μ*_Vol_	[day^–1^]	Vlume‐based growth rate

## CONFLICTS OF INTEREST

The authors have declared no conflicts of interest.

## Supporting information



Supporting Information

Supporting Figure S1

Supporting Figure S2

Supporting Figure S3
