# Supplementary material for: The cultivation conditions affect the aggregation and functionality of β‐cell lines alone and in coculture with mesenchymal stromal/stem cells
Source: Eng Life Sci. 2022 May 20;22(12):769–83. doi: 10.1002/elsc.202100168 (PMC9731603; doi:10.1002/elsc.202100168)
Supplement: Supplementary file 1 — Supporting Information [file ELSC-22-769-s001.docx]

# **Supplementary**

Table S1: Summarized key parameters of β-cell lines expanded as monolayers. We compared the three β-cell lines in terms of cell growth (growth rate µ_2D_ and doubling time t_D_) and insulin profile (basal/acute secretion, stimulation index SI) with other studies. However, direct comparison is challenging because different analytical methods were used and, most importantly, insulin secretion is often not normalized per cell.

| Cell type | Growth parameter | | Insulin secretion profile | | | Reference |
| --- | --- | --- | --- | --- | --- | --- |
|  | µ_2D_ [d^-1^] | t_D,min_ [d] | Basal | Acute | SI [-] |  |
| 1.1B4 | 0.63 ± 0.03 | 1.10 ± 0.05 | - | - | - | This study |
|  | ~0.7 | ~1 d | 0.12 ng (10^6^ cell h)^-1^ | 0.27 ng (10^6^ cell h)^-1^ | 2.3 | [10] |
| INS-1 | 0.63 ± 0.10 | 1.11 ± 0.18 | 47 ± 20  ng (10^6^ cell h)^-1^ | 95 ± 19  ng (10^6^ cell h)^-1^ | 2.1 ± 0.9 | This study |
|  | - | - | 8.40 ± 1.66 ng mL^-1^ | 72.19 ± 10.69 ng mL^-1^ | 8 | [23] |
| INS-1 832/13 | 0.38 | 1.8 | ~200 µU (mg h) | ~1900 µU (mg h) | 9.2 | [62] |
| MIN6 | 0.35 ± 0.03 | 2.00 ± 0.17 | 166 ± 15  ng (10^6^ cell h)^-1^ | 985 ± 156  ng (10^6^ cell h)^-1^ | 6.0 ± 1.5 | This study |
|  | 0.2 | 2.98 ± 0.06 h | 0.56 ± 0.05 ng (µg protein h)^−1^ | 1.44 ± 0.19 ng (µg protein h)^−1^ | 2.5 | [35] |
| EndoC-βH1 | 0.1 | 7.25 | ~5200 µU (mg h) | ~12,500 µU (mg h) | 2.4 | [62] |


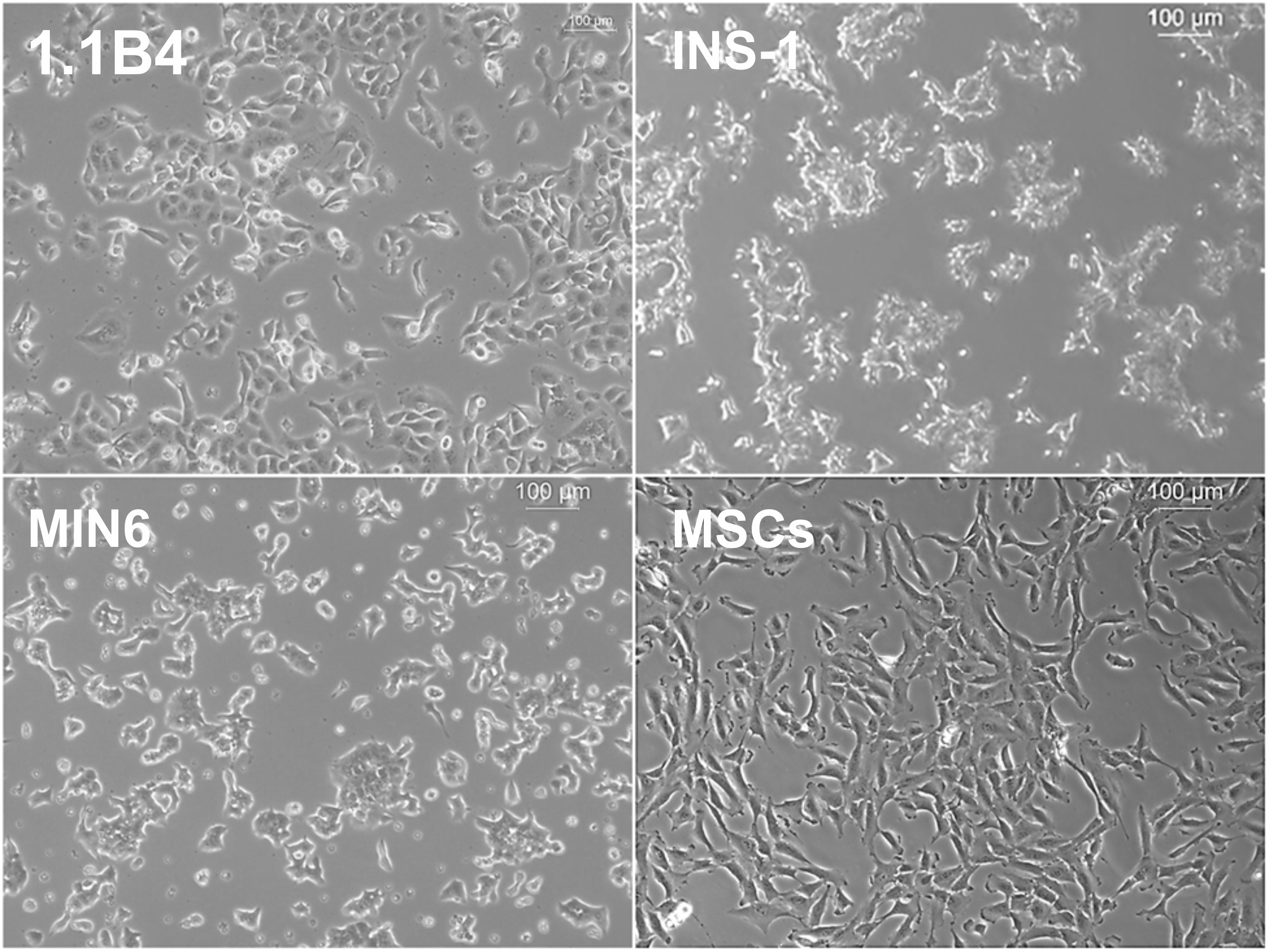


Figure S1: The three β-cell lines were morphologically distinct. The human 1.1B4 cells (upper left) were the largest, forming a distinct monolayer with an epithelial morphology, whereas the rodent INS-1 (upper right) and MIN6 cells (bottom left) were smaller and formed clusters. Both MSCs (ad-MSCs and hMSC-TERTs) showed a fibroblast-like morphology.


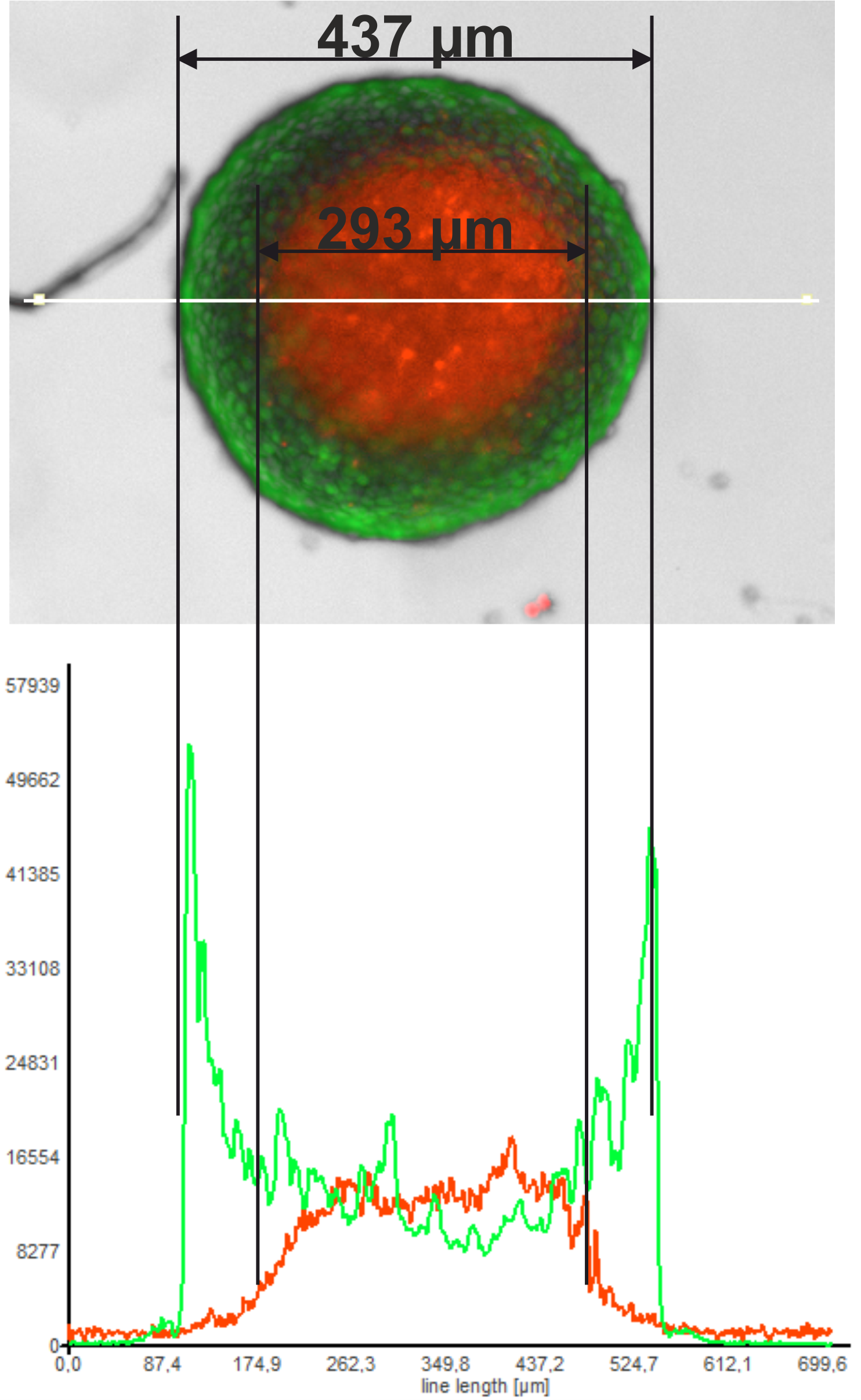


Figure S2: Based on the “Line Profile”, the diffuse distance of the spheroids was determined by fluorescence staining. We assume that the zone of viable cells is directly correlated to the presence of enough nutrients which diffuse into the spheroids. As the cells consume nutrients over this distance, the end of this distance is given by the concentration of one main metabolite (mostly oxygen) being zero. We measured the distance (radius) of the viable outer layer to the dead spheroid core as seen here for the INS-1 cells.


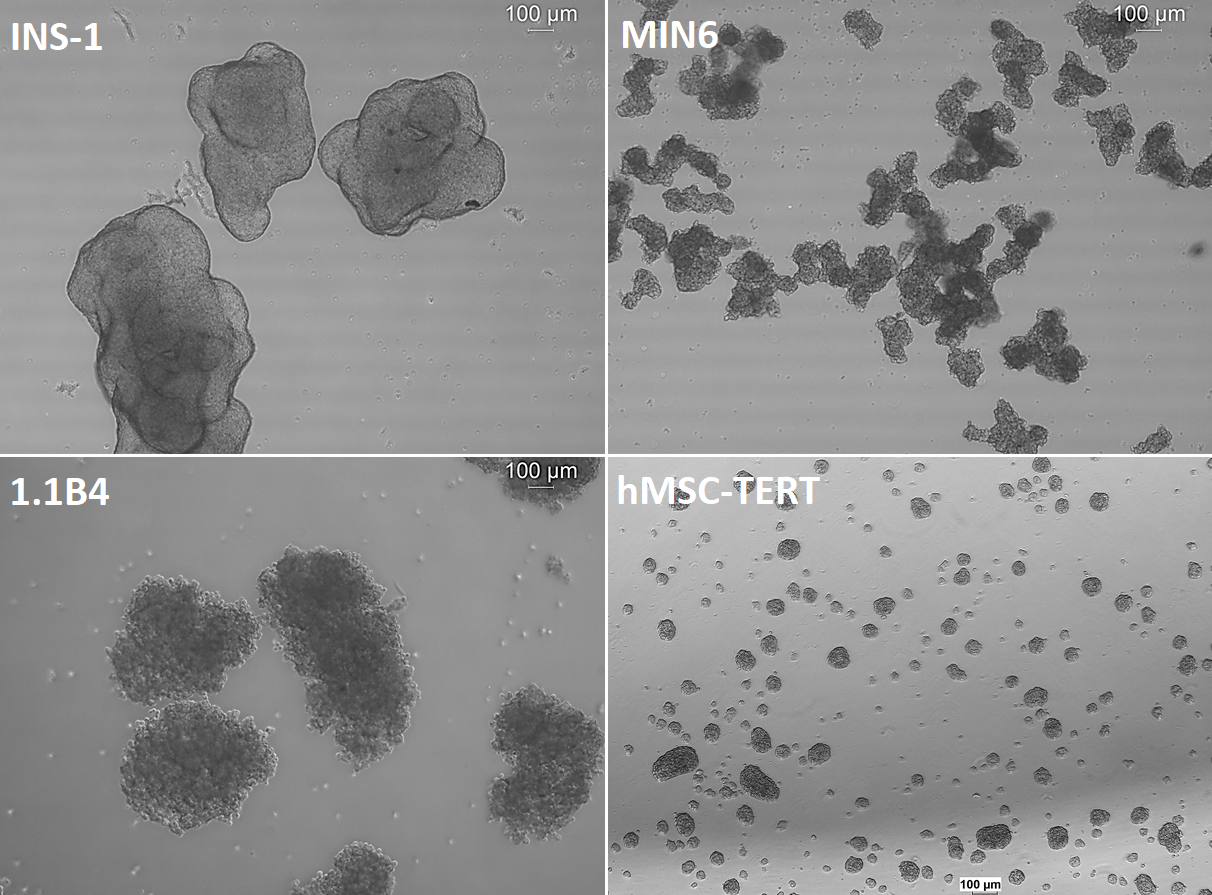


Figure S3: Representative images of all β-cell lines and the hMSC-TERT line isolated from shaken 12-well plates after 1 d, which served as basis for further dynamic culture systems.
