# Supplementary figures and images for: The cultivation conditions affect the aggregation and functionality of β‐cell lines alone and in coculture with mesenchymal stromal/stem cells
Source: Eng Life Sci. 2022 May 20;22(12):769–83. doi: 10.1002/elsc.202100168 (PMC9731603; doi:10.1002/elsc.202100168)

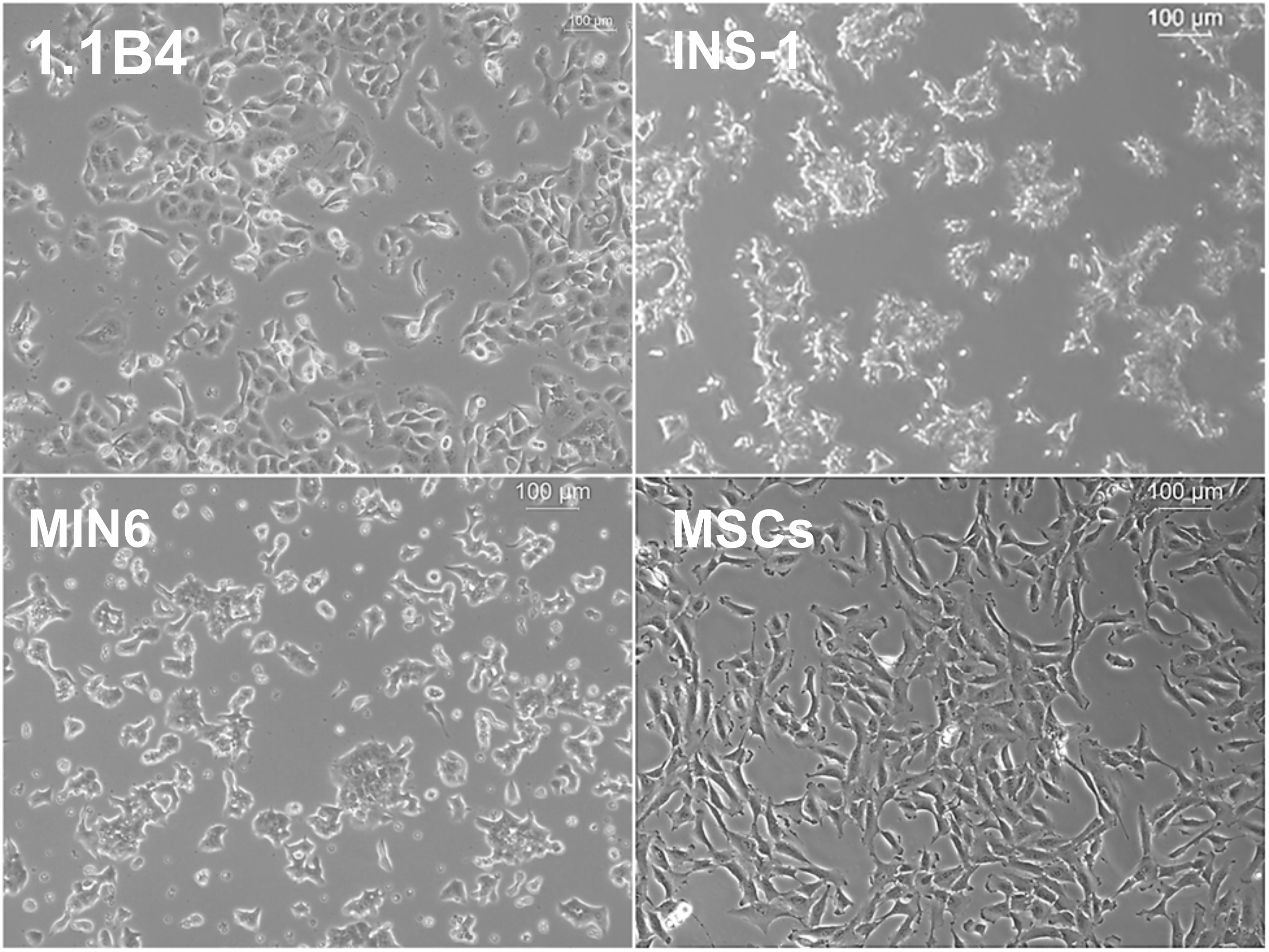

Supplement: Supplementary file 2 — Supporting Figure S1 [file ELSC-22-769-s004.png]

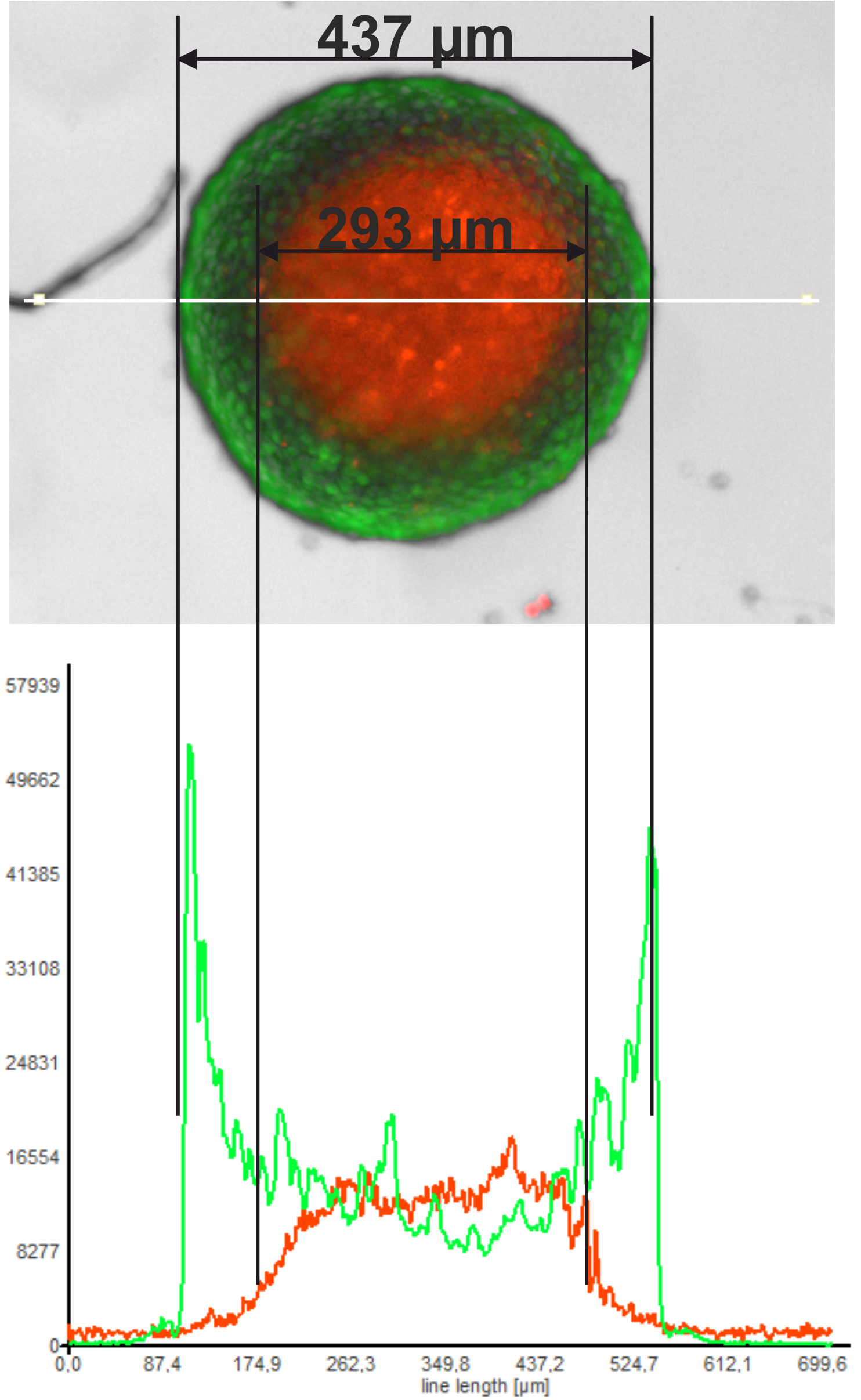

Supplement: Supplementary file 3 — Supporting Figure S2 [file ELSC-22-769-s003.png]

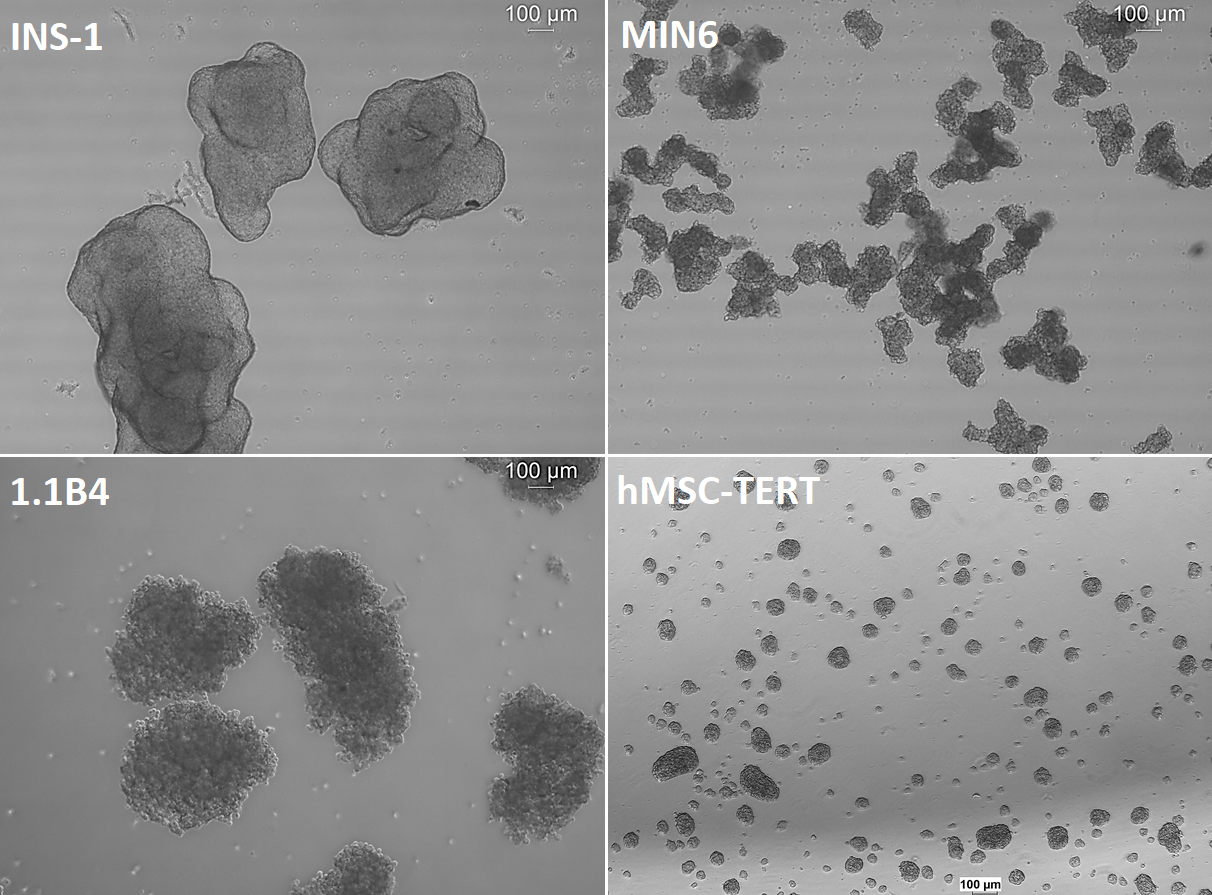

Supplement: Supplementary file 4 — Supporting Figure S3 [file ELSC-22-769-s002.png]
